# *Scabiosa stellata* L. Phenolic Content Clarifies Its Antioxidant Activity

**DOI:** 10.3390/molecules23061285

**Published:** 2018-05-27

**Authors:** Naima Rahmouni, Diana C. G. A. Pinto, Noureddine Beghidja, Samir Benayache, Artur M. S. Silva

**Affiliations:** 1 Campus de Santiago, Department of Chemistry & QOPNA, University of Aveiro, 3810-193 Aveiro, Portugal; rahmouni_na@yahoo.fr (N.R.); diana@ua.pt (D.C.G.A.P.); 2 Unité de Recherche et Valorisation des Ressources Naturelles, Molécules Bioactives et Analyse Physico-chimiques et Biologiques, Université des Frères Mentouri Constantine 1, Constantine, Algeria; nourbeghidja@yahoo.fr (N.B.); sbenayache@yahoo.com (S.B.)

**Keywords:** *Scabiosa stellata* L., phenolic profile, antioxidant activity, UHPLC-MS, NMR, flavone glycosides

## Abstract

The phenolic profile of *Scabiosa stellata* L., a species used in Moroccan traditional medicine, is disclosed. To obtain that profile the species extract was analyzed by ultra-high-performance chromatography coupled to photodiode-array detection and electrospray ionization/ion trap mass spectrometry (UHPLC-DAD-ESI/MS^n^). Twenty-five phenolic compounds were identified from which isoorientin and 4-*O*-caffeoylquinic acid can be highlighted because they are the major ones. The antioxidant activity was significantly controlled by the fraction type, with the *n*-butanol fraction showing the highest antioxidant activity (FRS_50_ = 64.46 µg/mL in the DPPH assay, FRS_50_ = 27.87 µg/mL in the ABTS assay and EC_50_ = 161.11 µg/mL in the reducing power assay). A phytochemical study of the *n*-butanol fraction was performed, and some important flavone glycosides were isolated. Among them the tamarixetin derivatives—the less common ones—can be emphasized. This phytochemical study and polyphenolic profile can be correlated with *S. stellata* extracts in vitro antioxidant activity. Moreover, it can be regarded as an evidence of its medicinal use and can incentivize its consumption.

## 1. Introduction

It has been recognized that two-thirds of the word’s plant species have medicinal value. Botanical preparations used in folk medicine for multiple purposes are being increasingly studied. Furthermore, in a society concerned with health and nutrition, these natural sources are emerging as a strong alternative in pharmaceutical and nutritional fields [1,2,3,4].

The genus *Scabiosa*, mainly distributed in Southern Africa, Europe and Asia [5,6], is the most significant member of the family Caprifoliaceae and, accordingly to the Plant List data base, incorporating 72 accepted species [7], from which 11 grow wildly in Algeria and have been used in folk medicine [8]. (This genus species are currently placed in the family Caprifoliaceae; however in former publications the genus was placed in the Dipsacaceae family).

Regarding *Scabiosa stellata* L., and as far as we are aware of, only three studies involving its chemical composition were recently reported [9,10,11], its medicinal use in traditional medicine was also reported [12] but has not yet been validated. These previous phytochemical studies revealed the presence of important secondary metabolites, such as fatty acids and triterpenoids [9,11], among which several are new triterpenoid saponins [11]. Furthermore, new bis-iridoids and three known flavonoids were also described [10]. Important biological activities were recently reported [10], among which the antioxidant activity of a 70% EtOH extract (In the table of the original manuscript, is indicated that the extract evaluated is a 70% MeOH. However, we think it was a mistake because in the experimental section is indicated a 70% EtOH)a fact that is important because increasing evidence has shown that antioxidant activity of plants is often related to its individual phenolic compounds [13,14,15]. The innovative character of studying *S. stellata*, a species whose chemical profile is not entirely established, is also relevant due to its high dissemination in the Algerian territory. With the aim of correlating the *S. stellata* phytochemicals with the antioxidant response of its extracts, a bio-guide phytochemical study was performed. In addition, as far as we know, this is the first study to report a detailed characterization in phenolic compounds of *S. stellata*, and point out to the nutritional value of this species.

## 2. Results and Discussion

**Figure 1:**
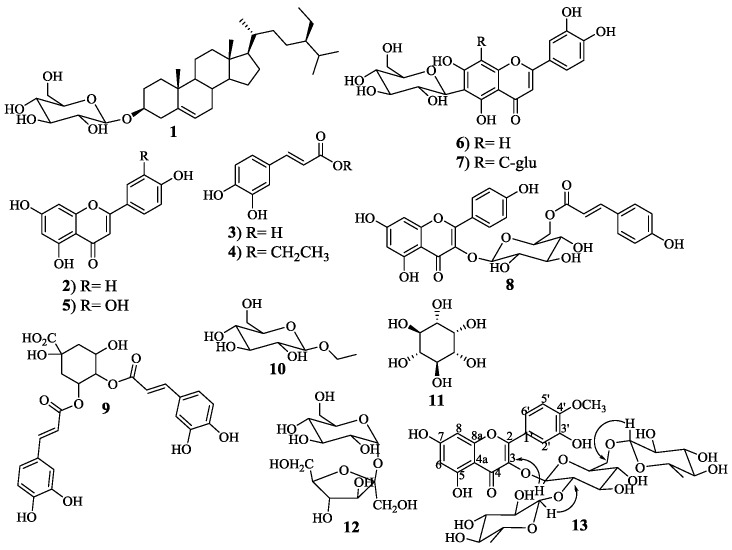
Structures of isolated compounds: β-sitosterol-β-d-glucoside **1**, apiginin **2**, caffeic acid **3**, ethyl caffeate **4**, luteolin **5**, isoorientin **6**, lucenin 2 **7**, tiliroside **8**, 4,5-*O*-dicaffeoylquinic acid **9**, 1-*O*-ethyl-β-d-glucoside **10**, myo-inositol **11**, β-d-fructofuranosyl-(2→1)-α-d-glucoside **12** and tamarixetin 3-β-l-rhamnosyl-(1→2)[β-l-rhamnosyl-(1→6)]β-d-glucoside] **13**.

Aiming to establish the phenolic profile of *S. stellata*, an ethanolic extract was obtained and fractioned in dichloromethane fraction (DCMF), ethyl acetate fraction (EAF) and *n*-butanol fraction (*n*-BF). Considering that this species’ biological activity [10] should be more related to the phenolic content, a previous determination of fractions total phenolic content was performed as well as their antioxidant activity (Table 1). These determinations allowed confirmation that the fraction obtained with dichloromethane, although more representative than the one obtained with ethyl acetate (more than two times higher), is less rich in phenolic compounds. This low content in phenolic compounds also explains why FRS_50_ (free radical scavenge) and EC_50_ values were not found for this fraction of antioxidant assays (Table 1). The antioxidant potential of these fractions was estimated by three in vitro assays and the results are shown in Table 1. DPPH and ABTS assays measure the abilities of the fractions to scavenge free radicals while reducing power assay evaluate their ability to reduce Fe^3+^ to Fe^2+^, in all cases it is recognized that the phenolic compounds are responsible for these antioxidants abilities [16]. Therefore, the gathered data allowed inferring some general conclusions regarding the antioxidant activity of each fraction. The DCMF has no significant activity, which is in accordance with its low content in phenolic compounds. The *n*-BF is the more active one in all the assays, which is also in harmony with its higher content in phenolic compounds. However, the overall results are considerably higher than the used references it seems that *S. stellata* possess antioxidant metabolites that can be further evaluated. Concerning antioxidant activity of this species, the only data reported so far involves the DPPH assay [10]. The herein reported results are similar to the previous ones, moreover the two extra assays herein performed also corroborate that antioxidant active metabolites can be produced by this species. Furthermore, there is evidence that other *Scabiosa* species extracts have antioxidant active [17,18]. These bioassays guide us to perform the phytochemical study of the EAF and *n*-BF fractions. The total phenolic content of both fractions (Table 1) explains the fact that more phenolic compounds were isolated from the *n*-BF fraction (Figure 1 and experimental section).

**Figure 2:**
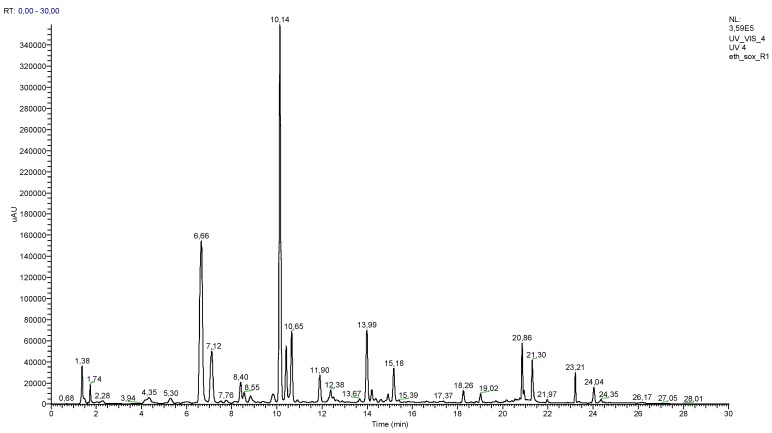
UHPLC chromatogram of *S. stellata* ethanolic extract recorded at 305 nm.

Another significant result that can be noticed from the data in Table 1 is the fact that these solvents only extracted 69.3% of the original ethanolic extract. To obtain a more accurate phenolic profile of *S. stellata*, the ethanolic extract was screened by UHPLC-DAD-ESI/MS^n^ analysis and the UV chromatogram, recorded at 305 nm (Figure 2). A careful analysis of the chromatogram revealed two major peaks eluted at 6.66 and 10.14 min and several other minor peaks (Figure 2). Twenty-five phenolic compounds could be identified and, from those, nine are chlorogenic acid derivatives and thirteen are flavonoid derivatives (data of the retention time, maximum wavelength, molecular ions species and fragments are presented in Table 2), and represent, respectively, 30% and 56% of the total phenolic amount. The fact that these types of compounds are recognized as antioxidants [16,19] is consistent with the recently reported [10] and above-mentioned antioxidant activity.

The nine chlorogenic acids herein identified as constituents of the *S. stellata* ethanolic extract were mainly identified through their pseudomolecular ions ([M − H]^−^) and MS^n^ fragments. In the case of 4,5-*O*-dicaffeoylquinic acid (isochlorogenic acid C) **9** (Figure 1; Table 2 Rt = 13.99 min), which was isolated and characterized, the confirmation was also obtained by the injection of the pure compound. In fact, the literature is rich in these acids’ MS data due to their ubiquitous occurrence in plants [15,20,21,22,23,24,25,26,27,28,29] and consequently, peaks at 4.35, 6.66, 7.12 and 8.55 min with [M − H]^−^ at *m*/*z* 353 were identified as 1-*O*-caffeoylquinic, 4-*O*-caffeoylquinic, 3-*O*-caffeoylquinic and 5-*O*-caffeoylquinic acids, respectively. The 4-*O*-caffeoylquinic acid is easily distinguished from the others due to its characteristic MS^2^ base peak at *m*/*z* 173, whereas in the other derivatives the base peak in MS^2^ is at *m*/*z* 191 (Table 2). On the other hand, 3-*O*-caffeoylquinic acid could be assigned to be the peak at 7.12 min due to the absence of the fragment ion at *m*/*z* 135 in the MS^2^ spectra characteristic in the others caffeoylquinic acids (Table 2). The other two monosubstituted quinic acids, peaks at 8.83 and 9.83 min, were identified respectively as 5-*O*-*p*-coumaroylquinic acid and 5-*O*-feruloylquinic acid due to their [M − H]^−^ at *m*/*z* 337 and *m*/*z* 367 (Table 2). Furthermore, the base peak in MS^2^ at *m*/*z* 191 allowed us to distinguish the 5-*O*-*p*-coumaroylquinic acid from the other possible isomers [22]. The analysis of the MS^3^ spectra allowed other assignments and confirmation of the abovementioned data.

Disubstituted quinic acids were another type of chlorogenic acids found in the extract, and correspond to the peaks at 13.99, 14.38 and 15.18 min, all with [M − H]^−^ at *m*/*z* 515 (Table 2). Peaks at 14.38 and 15.18 min could be assigned, respectively to 3,4-*O*-dicaffeoylquinic and 3,5-*O*-dicaffeoylquinic acids, due to the base peak in MS^3^ spectra (respectively *m*/*z* 173 and *m*/*z* 191). In the case of peak at 13.99 min the phytochemical study of the extract allowed the isolation and full characterization of 4,5-*O*-dicaffeoylquinic acid **9** (Figure 1), which could be used as standard and confirm the identification of this peak. It is interesting to notice that *S. stellata* areal parts are rich in these important metabolites [19], particularly in 4-*O*-caffeoylquinic acid, which accounts for 46% of the total chlorogenic acids content (Table 2). Most of these compounds are herein reported for the first time in this species and the earlier reported presence of 3,5-*O*-dicaffeoylquinic acids and 4,5-*O*-dicaffeoylquinic acid [10] is also confirmed.

The phytochemical study allowed the isolation of caffeic acid and its derivatives as well as several glucosides (Figure 1), confirming the presence of other caffeic acid derivatives in the extract. Some of the isolated compounds were not detected by UHPLC-DAD-ESI/MS^n^, from which compound **1** (Figure 1) should be highlighted. The compound was afterwards injected, and it could be detected at another wave length (250 nm) but not the one used to perform the analysis herein discussed. The same was verified in the case of the ethyl derivatives isolated (compounds **4** and **10**, Figure 1). Additionally, we cannot confirm that they are natural derivatives; in fact, they can be formed during the extraction with ethanol.

The ethanolic extract profile of *S. stellata* shows the presence of another important family of secondary metabolites, the flavonoids, for which literature is also rich in MS data [24,25,30,31,32,33,34,35,36] and the presence of isoorientin, hyperoside and swertiajaponin in *S. stellata* extract was recently reported [10]. The detailed analysis of the characteristic MS^n^ fragment ions (Table 2), as well as the phytochemical study, allowed the identification of several flavonoid derivatives, which in fact represent 56% of the total phenolic amount. The previous reported isoorientin and hyperoside [10] were also found together with several others that are herein reported for the first time in this species. The main aglycones found are flavone (apigenin, diosmetin and luteolin) and flavanol (kaempferol, quercetin and tamarixetin) types. Most of the identified compounds are known; in fact, they are secondary metabolites ubiquitous in nature. Moreover, the results herein reported are identical to the ones reported in the literature [24,32,33,34,35]. In all cases the ion fragment with *m*/*z* value of the key aglycone is the base peak of MS^2^ or MS^3^ (Table 2), making easier their identification. Luteolin glycosides are the major constituents and account for 74% of the total flavonoid content (Table 2). Moreover, luteolin-6-*C*-glucoside, which elutes at 10.14 min is the major constituent of the *S. stellata* ethanolic extract (34%).

Tamarixetin glycosides are less reported in the literature and in the *S. stellata* extracts, as far as we are aware, this is the first report on its occurrence. Three derivatives were detected, the peaks eluted at 14.93, 20.86 and 20.94 min. The peak eluted at 14.93 min correspond to extremely small amount of tamarixetin-*O*,*O*-dihexoside, the [M − H]^−^ at *m*/*z* 639 and the base peak at *m*/*z* 315 in MS^2^ suggest it. The peak eluted at 20.86 has similar absorption in the UV-Vis region and the base peak at *m*/*z* 315 in MS^2^ confirming that it should be a tamarixetin derivative (Table 2) and in this case in a considerable amount (nearly 7%). A careful analysis of the chromatogram (Figure 2) allowed detection of the peak eluted at 20.94 min with [M − H]^−^ at *m*/*z* 769 and the base peak at *m*/*z* 315 in MS^2^; due to its proximity with the peak at 20.86 min, the quantification was not possible (Table 2). However, the similarity of this peak data and the above-mentioned ones suggests that it is also a tamarixetin derivative. The phytochemical study allowed the isolation and characterization of several flavonoids and their glycosides (Figure 1) which confirmed the above-discussed identification. In the case of the peak eluted at 20.94 min, the confirmation was also established because this tamarixetin glycoside **13** (Figure 1) was isolated and fully characterized. At first glance, this compound seems to be an unusual tamarixetin derivative, whose occurrence in nature was recently reported and not in the *Scabiosa* genus [37]. Therefore, to confirm the identification its characterization was meticulous and compared with the previous reported data and accordingly the compound was identified as being the tamarixetin 3-β-l-rhamnosyl-(1→2)β-l-rhamnosyl-(1→6)]β-d-glucoside] **13** (Figure 1). First, the aglycone nucleus was assigned due to the characteristic signals in the aromatic region at δ 6.21 and 6.41 ppm, correspond, respectively to the resonance of protons H-6 and H-8. The signal shape, doublets with a coupling constant *J* = 2.1 Hz (characteristic of a *meta* position coupling), is consistent with the tamarixetin substitution pattern (Figure 1). Moreover, the ring B substitution pattern was established due to the singlet at δ 3.98 ppm confirming the presence of a methoxy group, the two doublets at δ 7.96 ppm (*J* = 2.1 Hz) and δ 6.94 ppm (*J* = 8.4 Hz) respectively to the resonance of protons H-2′ and H-5′ and a double doublet at δ 7.60 ppm (*J* = 8.4 and 2.1 Hz) assigned to H-6′, which confirms its *ortho*-coupling with H-5′ and meta-coupling with H-2′.

The presence of the ^13^C-NMR characteristic signals (Table S1) also proves that the aglycone is tamarixetin; moreover, these results are in accordance with the previous data [37]. The glycoside residue was also established, with the presence of three doublets at δ 5.75, 5.21 and 4.56 ppm, characteristic signals of anomeric protons, indicative of the three hexoses. The two doublets at δ 1.08 and 0.93 ppm, typical of methyl groups, confirm that two are rhamnoses and the signal at δ 5.75 ppm confirms the presence of glucose. In the HMBC can be noticed, among others, the correlation between the anomeric proton of the glucose unit and the tamarixetin carbon C-3, the correlation between the anomeric protons of one of the rhamnose unit with the glucose carbon C-6 and the other with the glucose carbon C-2 (Figure 1). The obtained data confirm not only the glycosylic residue but also its linkage to the aglycone moiety as depicted in Figure 1.

Tiliroside **8** (Figure 1) is another important flavonoid found for the first time in this genus. It is a kaempferol derivative, the kaempferol 3-*O*-β-d-glucopyranoside-6-*p*-coumaryl ester, for which biological significance was reported [38], and was isolated but also found in the UHPLC-MS profile. It is the peak eluted at 19.02 min (Table 2).

## 3. Materials and Methods

### 3.1. Chemicals

Pure compounds were used as standards to elucidate the identification of the phytochemicals and to elaborate the calibration curves following the external standard method. Moreover, the isolated compounds were also used as standards. The phenolic standards benzoic acid, cyanuric acid, 4-hydroxy-3-methoxybenxoic acid, gallic acid, sinapic acid, catechin, rosmarinic acid, isorhamnetin, kaempferol, luteolin, diosmetin and quercetin were purchased from Sigma-Aldrich (St. Louis, MO, USA). The phenolic standards 3,4-dihydroxycinnamic acid (caffeic acid) and 4-hydroxycinnamic acid (p-coumaric acid) were purchased from Acros Organics (Geel, Belgium). The phenolic standards ferulic acid and chlorogenic acid were purchased from Extrasynthese (Genay Cedex, France). Solvents were purchased from Panreac and Acros Organics and were of HPLC purity, analytical grade, or bi-distilled commercial solvents. Chromatographic purifications were performed using silica gel 60 (70–230 mesh, Merck Kieselgel, Kenilworth, NJ, USA), Sephadex LH-20 and Merck silica gel 60 GF254. Iron(II) sulphate, potassium hexacyanoferrate(III), iron(III) chloride, butylated hydroxyanisole (BHA), ascorbic acid, Folin & Ciocalteu′s phenol reagent, trichloroacetic acid (TCA), 2,2-diphenyl-1-picrylhydrazyl (DPPH), 2,2′-azino-bis(3-ethylbenzothiazoline-6-sulfonic acid) solution (ABTS solution) and 6-hydroxy-2,5,7,8-tetramethylchroman-2-carboxylic acid (trolox) were obtained from Sigma-Aldrich (St. Louis, MO, USA).

### 3.2. Plant Collection and Extract Preparation

The whole plant of *Scabiosa stellata* L. was collected in June 2015 from Batna [the specimens of *S. stellata* were collected in the Belezma National Park (Batna, Algeria; 35°35′41.52″ N, 5°56′13.75″ E)]. A voucher specimen was identified by Dr. Bachir Oudjehih professor of Agronomic Institute, University of Batna under the reference number VAREN/SS/2013/123.

Dried-air powdered of *S. stellata* all plant (500 g) was macerated firstly with *n*-hexane to eliminate fatty acids and other lipophilic components, then was extracted with ethanol (6 L, 2 days cycles and three times) using sohxlet. The mixture was filtered, and the combined extracts were concentrated under vacuum giving 88.6 g. Then, the extract was sequential dissolved by different solvents (increasing polarities). After filtration and evaporation 10.9 g of dichloromethane fraction (DCMF), 4.7 g of ethyl acetate fraction (EAF) and 45.8 g of *n*-butanol fraction (*n*-BF) were obtained.

### 3.3. UHPLC-DAD-ESI/MS^n^

For the UHPLC-MS analysis, 50 mg of each extract were dissolved in 5 mL of methanol (final concentration 10 mg/mL) and the resulting solutions were filtered through a 0.2 mL nylon membrane (Whatman, Maidstone, UK). Three independent analyses were carried out for reproducibility. This technique was performed using a Thermo Scientific Ultimate 3000RSLC (Dionex, Sunnyvale, CA, USA) equipped with a Dionex UltiMate 3000 RS diode array detector and coupled to a mass spectrometer. The column used was a Thermo Scientific hypersil gold column (Part n° 25002-102130; Dim 100 mm × 2.1 mm; Lot 14913; SN 10518298) with a part size of 1.9 µm and its temperature was maintained at 30 °C. The mobile phase was composed of (B) acetonitrile and (A) 0.1% formic acid in water (*v*/*v*), both degassed and filtered before use. The flow rate was 0.2 mL/min. The elution gradient was 5% (solvent A) for 14 min, 40% (solvent A) over 2 min, 100% (solvent A) over 7 min and the re-equilibration of the column with 5% of solvent A for 10 min. The injection volume was 2 µL. UV-vis spectral data were gathered in a range of 250 to 500 nm and the chromatographic profiles were documented at 280 nm. The mass spectrometer used was an LTQ XL linear ion trap 2D equipped with an orthogonal electrospray ion source (ESI). The equipment was operated in negative-ion mode with electrospray ionization source of 5.00 kV and ESI capillarity temperature of 275 °C. The full scan covered a mass range of 50 to 2000 *m*/*z*. Collision-induced dissociation MS/MS and MS^n^ experiments were simultaneously acquired for precursor ions.

### 3.4. Phytochemical Analysis

EAF was separated over column chromatography using silica gel and a gradient elution starting with hexane/ethyl acetate (100:0 to 0:100) and followed by ethyl acetate/methanol (100:0 to 0:100). Several fractions were obtained and compound **1** (Figure 1) was isolated pure. Compound **2** (Figure 1) was isolated from the less polar fractions by thin-layer chromatography and using hexane/ethyl acetate (85:15) as eluent. More polar fractions were purified by column chromatography using sephadex LH20 and eluted with methanol and allowed the isolation of compounds **3** and **4** (Figure 1). The detailed scheme is available in Supplementary Material (Figure S1).

*n*-BF was separated over column chromatography using silica gel and a gradient elution with chloroform/methanol (100:0 to 50:50). Twenty-five fractions were obtained and from their purification several other phenolic compounds were obtained. For example, from the first four fractions and by purification with column chromatography using sephadex LH20 and eluted with chloroform/methanol mixtures were obtained the pure compounds **5**, **6**, **7** and **8** (Figure 1). From fraction twelve was obtained after column chromatography using silica gel and eluting with a gradient of dichloromethane/methanol (100:0 to 0:100) compound **9** (Figure 1). From fraction fourteen and using a sephadex LH20 column chromatography was obtained compound **10** (Figure 1). Fractions eleven and fifteen allowed respectively the isolation of compound **11** and **12** (Figure 1). Finally, fraction twenty gave the pure compound **13** (Figure 1). The detailed scheme is available in Supplementary Material (Figure S2).

### 3.5. Identification and Quantification of the Phenolic Compounds

The quantification of the total phenolic content was carried out through Folin-Ciocalteu method [15] with some modifications. In a 96 well-microplate, 15 μL of a solution of each fraction was added to 60 μL of milliQ water and 15 μL of Folin-Ciocalteu reagent. After 5 min, 150 μL of a 7% Na_2_CO_3_ solution was added and the mixture was homogenized and incubated in the dark at 30 °C for 60 min. The absorbance was measured at 700 nm and the total phenolic content was expressed as a function of a linear calibration curve performed by a standard (gallic acid at different concentrations, 0.001 to 0.01 μg/mL). These contents are expressed in mg of gallic acid (GA) per gram of dry plant (DF) extract (mg GA.g^−1^ DP) and presented in Table 2.

The identification of individual phenolic compounds in the UHPLC analysis was achieved by comparison of their retention times, UV-Vis spectra, and MS^n^ spectra data with those of the closest available reference standards and data reported in the literature. In addition, the structure of some phenolic components was further confirmed by NMR analysis after their purification. The NMR spectra {^1^H, ^13^C, HSQC, HMBC [71 ms (7 Hz)], COSY} were measured in CDCl_3_, on a Bruker Avance 300 (300.13 MHz for ^1^H and 75.47 MHz for ^13^C) or Bruker Avance 500 with crioprobe (500.13 MHz for ^1^H and 125.76 MHz for ^13^C) spectrometers and using TMS as internal standard. Chemical shifts were reported in δ units (ppm) and coupling constants (*J*) in Hz.

The semi quantification of the main individual phenolic compounds in the extract was performed by peak integration at 260 nm, through the external standard method, using the closest reference compounds available. The detection and quantification limits (LOD and LOQ, respectively) were determined from the parameters of the calibration curves represented in Table S2 (LOD = 3 standard deviation/slope and LOQ = 10 standard deviation/slope). The calibration curves were obtained by injection of five known concentrations with variable ranges (Table S2) and the concentrations of the standards were chosen to guarantee the quantification of each compound in the samples by intrapolation in the calibration curve. Values of correlation coefficients confirmed linearity of the calibration plots (Table S2). The results were expressed in mg of compound/g of dried extract, as mean ± standard deviation of four independent analyses.

### 3.6. Evaluation of Antioxidant Activity

#### 3.6.1. DPPH Radical-Scavenging Assay

The radical-scavenging activity was carried out following a previously reported procedure [39], with some modifications. The reaction mixture in each one of the 96-wells consisted of one of the different concentrations of the fractions (50 μL) and 250 μL of a methanolic solution containing DPPH (0.2 mM). The microplates were then placed in the dark and at room temperature for 30 min. The absorbance was measured at 517 nm using a microplate reader (model EAR 400, Labsystems Multiksan MS) with reference to a control without extract. The radical-scavenging activity was calculated as a percentage of DPPH discoloration using the equation: DPPH scavenging effect % = [(A0 − A1)/A0)] × 100 where A0 is the absorbance of the control reaction and A1 is the absorbance of the test fraction. Based on graphic values of the inhibition percentage of DPPH vs. fraction concentration, the FRS_50_ of each extract was estimated (Table 2). Ascorbic acid was used as the reference.

#### 3.6.2. ABTS Assay

The ABTS assay was carried out following a previously reported procedure [40], with some modifications. A volume of 250 µL of ABTS solution (7 mM) was mixed with 50 μL of the plant extract fractions at various concentrations. The reaction mixture was left in the dark during 20 min, and its absorbance was recorded at 734 nm. As for the antiradical activity, ABTS scavenging ability was expressed as FRS_50_ (μg/mL) as shown in Table 2. Trolox was used as the reference.

#### 3.6.3. Reducing Power

The reducing power was determined according to a method described before [39]. This method consists to mixing 200 μL of the fraction at different concentrations with 200 μL of phosphate buffer (0.2 mM, pH 6.6) and 200 μL of a solution of K_3_Fe(CN)_6_ (1%). The obtained mixture was incubated for 20 min at 50 °C. Then 200 μL of TCA (10%) was added followed by vigorous stirring. Thereafter, 75 μL of each solution obtained was put in a well of the microplate and 75 μL of distilled water and 15 μL of FeCl_3_ (0.1%) were adjoined. Absorption reading was done at 690 nm (white is the extraction buffer). Based on the study of the change in absorbance as a function of the sample concentration, the results obtained to calculate the effective concentration (EC_50_, mg/mL; Table 2) which is the concentration of the corresponding sample at an absorbance of 0.5. BHA was used as the reference.

### 3.7. Statistical Analysis

Results were expressed as mean ± standard deviation of three independent assays and analyzed through unpaired Student’s test or ANOVA combined with Tukey’s test (Graph Pad Prism 5). P values of less than 5% (*p* < 0.05) were considered to be significant.

## 4. Conclusions

Using UHPLC-DAD-ESI/MS^n^, the knowledge of the *S. stellata* phenolic profile was successfully extended, for the first time establishing its content in important secondary metabolites, such as chlorogenic acids and flavonoids. Chlorogenic acids and flavonoids comprised more than 80% of the compounds found, which explains the recently reported antioxidant activity of this plant extract. Among the phenolic compounds herein reported, some for the first time in both the genus and species, both luteolin-6-*C*-glucoside and 4-*O*-caffeoylquinic acid can be highlighted because they are the major compounds. However, tamarixetin derivatives can also be emphasized, due to their detection for the first time in *Scabiosa* genus, in particular the tamarixetin 3-β-l-rhamnosyl-(1→2)[β-l-rhamnosyl-(1→6)]β-d-glucoside] whose structure confirmation requires more experiments. Furthermore, the presence of tiliroside also should be highlighted due to its importance in other plants. Therefore, the characterization herein reported will provide information about the *S. stellata* benefits to individuals and may contribute to increase its consumption, through infusions and/or condiments.

## Figures and Tables

**Table 1:** Extraction yields and antioxidant capacity of *S. stellata* ethanolic extract fractions.

Fraction	Mass ^a^	Total Phenolic Content ^b^	DPPH (FRS_50_) ^c^	ABTS Assay (FRS_50_) ^c^	Reducing Power (EC_50_) ^c^
DCMF	12.3	<1.00	>250	>250	>50
EAF	5.3	4.74 ± 0.01 *	71.82 ± 0.04 *	40.41 ± 0.02 *	202.41 ± 0.10 *
*n*-BF	51.7	11.86 ± 0.05 *	64.46 ± 0.01 *	27.87 ± 0.01 *^,#^	161.11 ± 0.08 *^,#^
Reference	-	-	8.21 ± 0.03 ^d^	12.07 ± 0.04 ^e^	18.03 ± 0.01 ^f^

Table Data represent the mean values ± SD of three independent assays performed in triplicate (*n* = 3). ^a^ % of dry weight. ^b^ mg GA/g DF. ^c^ μg/mL. ^d^ Reference used was ascorbic acid. ^e^ Reference used was trolox. ^f^ Reference used was BHA. * Statistically significant different with respect to the reference (Tukey’s test), *p* < 0.05. ^#^ Statistically significant different with respect to EAF (unpaired Student’s *t*-test), *p* < 0.05.

**Table 2:** Identification of UHPLC/DAD/ESI-MS^n^ data, and quantification of the most relevant compounds from the ethanolic extract of *S. stellata* (Retention time (Rt), wavelength of maximum absorption in the UV-Vis region (λmax), pseudomolecular and MS^n^ fragment ions, quantification (mean ± SD) and identification of the phenolic compounds).

Rt (min)	λmax	[M − H]^−^ (*m*/*z*) ^♦^	ESI-MS^2^; (MS^3^) (*m*/*z*) ^♠^	Quantity ^♣^	Compound
1.38	191, 267	387	341, 369; (179, 143, 161)	8.24 ± 0.03	1-Caffeoylglucose derivative ^(b)^
1.74	193, 202	128	85, 109	4.30 ± 0.02	Cyanuric acid ^(a)^
4.35	204, 324	353	191, 179, 135; (173, 127, 109)	nq	1-*O*-Caffeoylquinic acid ^(c)^
5.30	211, 278, 323	223	205, 115, 143, 159	0.26 ± 0.01	Sinapic acid ^(a)^
6.66	217, 298, 325	353	191, 179, 173, 135; (111, 93)	26.41 ± 0.30	4-*O*-Caffeoylquinic acid ^(c)^
7.12	216, 299, 325	353	191, 179; (173, 127, 85)	8.93 ± 0.12	3-*O*-Caffeoylquinic acid ^(a)^
8.40	206, 269, 348	609	489, 447, (357, 327, 285)	1.84 ± 0.03	Luteolin-6-*C*-glucoside-7-*O*-glucoside ^(b)^
8.55	199, 214, 270, 304	353	191, 179, 135; (173, 127, 85)	1.47 ± 0.01	5-*O*-Caffeoylquinic acid ^(c)^
8.83	220, 274, 310	337	191, 173; (127, 110, 93)	tr	5-*O*-*p*-Coumaroylquinic acid ^(c)^
9.83	230, 326	367	191; (173, 85)	0.97 ± 0.02	5-*O*-Feruloylquinic acid ^(c)^
10.14	209, 269, 350	447	429, 357, 327; (309, 297, 285)	66.31 ± 0.30	Isoorientin (luteolin-6-*C*-glucoside) ^(a)^
10.40	211, 269, 350	579	561, 447, 357, 327; (309, 297, 285)	9.78 ± 0.26	Luteolin-2″-*O*-pentosyl-6-*C*-hexoside ^(b)^
10.65	211, 270, 346	461	371, 341, 313; (299, 231)	13.97 ± 0.11	Diosmetin-6(or 8)-*C*-glucoside ^(b)^
11.90	225, 270, 338	563	443, 431; (311, 283, 269)	2.82 ± 0.01	Apigenin-2″-*O*-pentosyl-8-*C*-glucoside ^(b)^
12.38	232, 256, 353	463	301; (268, 179, 151)	0.97 ± 0.04	Quercetin-3-*O*-glucoside (hyperoside) ^(b)^
13.99	220, 241, 327	515	353; (191, 173)	16.03 ± 0.03	4,5-*O*-Dicaffeoylquinic acid ^(a)^
14.21	237, 267, 337	609	489, 369; (298, 285, 231)	1.23 ± 0.01	Lucenin 2 (luteolin-6,8-di-*C*-glucoside) ^(b)^
14.38	242, 326	515	353, 335; (173,111)	tr	3,4-*O*-Dicaffeoylquinic acid ^(c)^
14.93	240, 268, 314	639	616, 315	tr	Tamarixetin-*O*,*O*-dihexoside ^(b)^
15.18	242, 326	515	353; (191, 171, 127)	3.74 ± 0.02	3,5-O-Dicaffeoylquinic acid ^(c)^
18.26	239, 270, 351	613	489, 447, 429; (369, 309, 285)	0.43 ± 0.02	Luteolin-6-*C*-glucoside derivative ^(b)^
19.02	243, 267, 314	593	447, 285	0.37±0.02	Tiliroside ^(b)^
20.86	237, 267, 314	635	477, 315	14.49 ± 0.02	Tamarixetin derivative ^(b)^
20.94	237, 267, 313	769	623, 477, 315	nq	Tamarixetin glycoside ^(a)^
21.30	243, 269, 313	739	593, 447, 285	10.85 ± 0.01	Kaempferol-3-*O*-rutinoside derivative ^(b)^
				57.55 ± 0.11 ^♥^	Total chlorogenic acids
				108.20 ± 0.17 ^♥^	Total flavonoids

^♦^ molecular ion; ^♠^ main fragments; ^♣^ mg of compound/g dried extract; nq—not quantified; tr—traces; ^♥^ obtained by propagation. Compounds were identified by ^(a)^ comparison with pure standards, commercially available or isolated; ^(b)^ comparison with pure aglycone and literature data; ^(c)^ comparison with pure cinnamic acid derivative and literature data.

## Supplementary Materials

Table S1: Selection of the most significant chemical shift values found in the ^1^H- and ^13^C-NMR spectra (recorded in MeOH and 500 MHz) of the compound **13**, Table S2: Linearity (y = mx + b, where y corresponds to the standard peak area and x corresponds to the mass of standard), LOD and LOQ of pure compounds used as reference.
