# Supplementary material for: Scabiosa stellata L. Phenolic Content Clarifies Its Antioxidant Activity
Source: Molecules. 2018 May 27;23(6):1285. doi: 10.3390/molecules23061285 (PMC6100036; doi:10.3390/molecules23061285)
Supplement: Supplementary file 1 [file molecules-23-01285-s001.pdf]

Supplementary Material

Table S1. Selection of the most significant chemical shift values found in the ^{1}H and ^{13}C NMR spectra (recorded in MeOH and 500 MHz) of the compound **13**.

C_{number}	δ_C (ppm)	δ_H (ppm)
2	185.4	-
3	143.3	-
4	179.2	-
5	163.2	-
6	99.81	6.21 (d, *J* 2.1 Hz)
7	165.8	-
8	94.8	6.41 (d, *J* 2.1 Hz)
8a	158.6	-
4a	105.9	-
1’	123.3	-
2’	114.5	7.96 (d, *J* 2.1 Hz)
3’	148.4	-
4’	150.6	-
5’	116.1	6.94 (d, *J* 8.4Hz)
6’	123.7	7.60 (dd, *J* 2.1; 8.4 Hz)
glucose 1’’	100.5	5.75 (d, *J* 7.7 Hz)
2’’	80.1	3.67 (dd, *J* 7.7; 2.2 Hz)
3’’	78.8	3.58-3.64 (m)
4’’	73.9	3.58-3.64 (m)
5’’	77.2	3.32-3.35 (m)
6’’	68.1	3.45-3.48(m)<br>3.86 (dd, *J* 11.4; 1.6 Hz)
rhamnose 1’’’	102.5	5.21 (d, *J* 1.3 Hz)
2’’’	72.0	3.98-4.02 (m)
3’’’	72.3	3.45-3.49 (m)
4’’’	73.9	3.30-3.33 (m)
5’’’	69.9	4.06 (dq, *J* 9.8; 6.2 Hz)
6’’’	17.4	0.93 (d, *J* 6.2 Hz)
rhamnose 1’’’’	102.8	4.56 (d, *J* 1.5Hz)
2’’’’	72.1	3.59-3.62 (m)
3’’’’	72.3	3.45-3.49 (m)
4’’’’	73.8	3.22-3.27 (m)
5’’’’	69.8	3.38-3.43 (m)
6’’’’	17.8	1.08 (d, *J* 6.2 Hz)

OCH_3	57.0	3.98 (s)

Table S2. Linearity (y = mx + b, where y corresponds to the standard peak area and x corresponds to the mass of standard), LOD and LOQ of pure compounds used as reference

Standard compound	Range concentration §	Slope (m) §§	Intercept (b) §§	R^2	LOD §	LOQ §
Benzoic acid	0.5-500	16748	111	1.0000	12	40
Gallic acid	0.5-500	557	-728	0.9988	11	37
Catechin	0.5-250	142	-58	0.9997	8	27
Caffeic acid	0.5-550	618	-70	0.9998	14	47
p-Coumaric acid	0.5-550	716	122	0.9990	15	50
Ferulic acid	0.5-500	1633	6	0.9993	10	33
Rosmarinic acid	0.5-250	706	1228	0.9976	7	23
Chlorogenic acid	0.5-250	659	-8	0.9989	9	30
Isorhamnetin	0.5-100	629	-2316	0.9991	3	10
Kaempferol	0.5-175	792	-76	0.9969	5	17
Luteolin	0.5-100	354	-221	1.0000	3	10
Quercetin	0.5-175	317	-3	0.9992	4	13

§in µg/mL
§§in area counts/mg

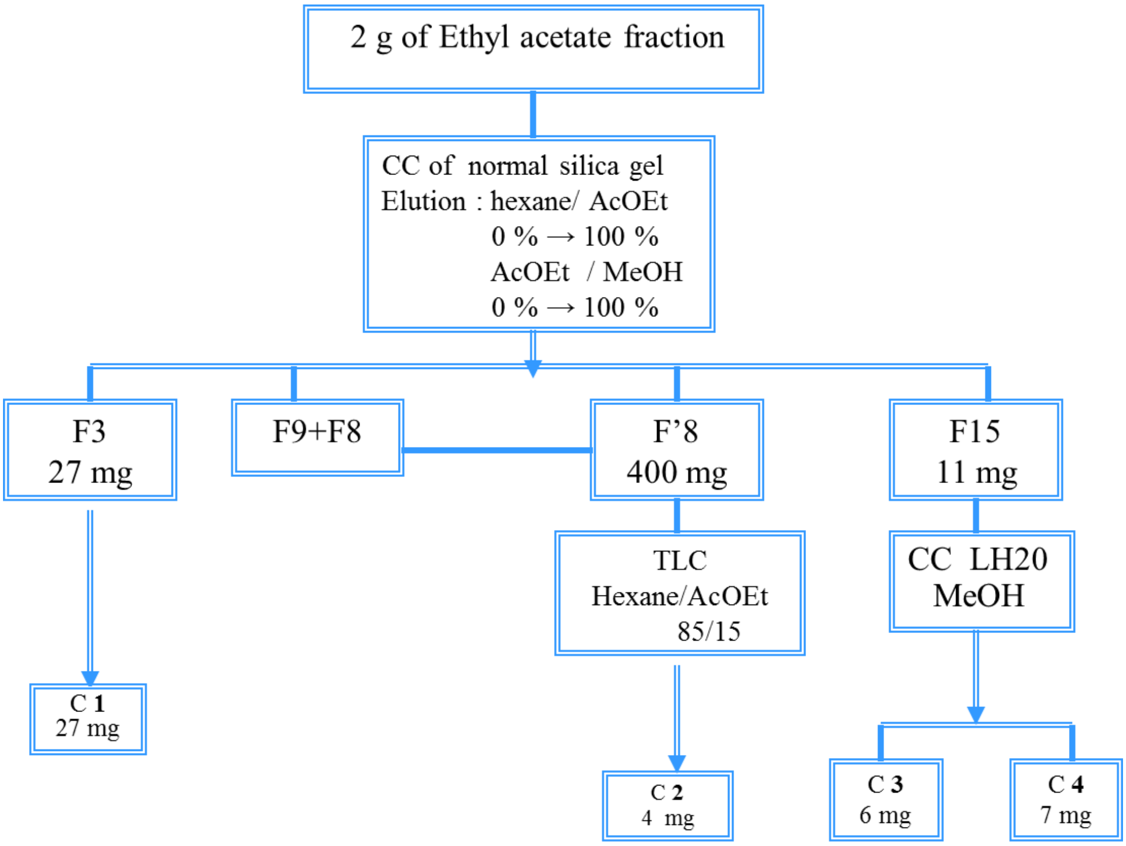

Figure S1. Scheme of the ethyl acetate fraction purification

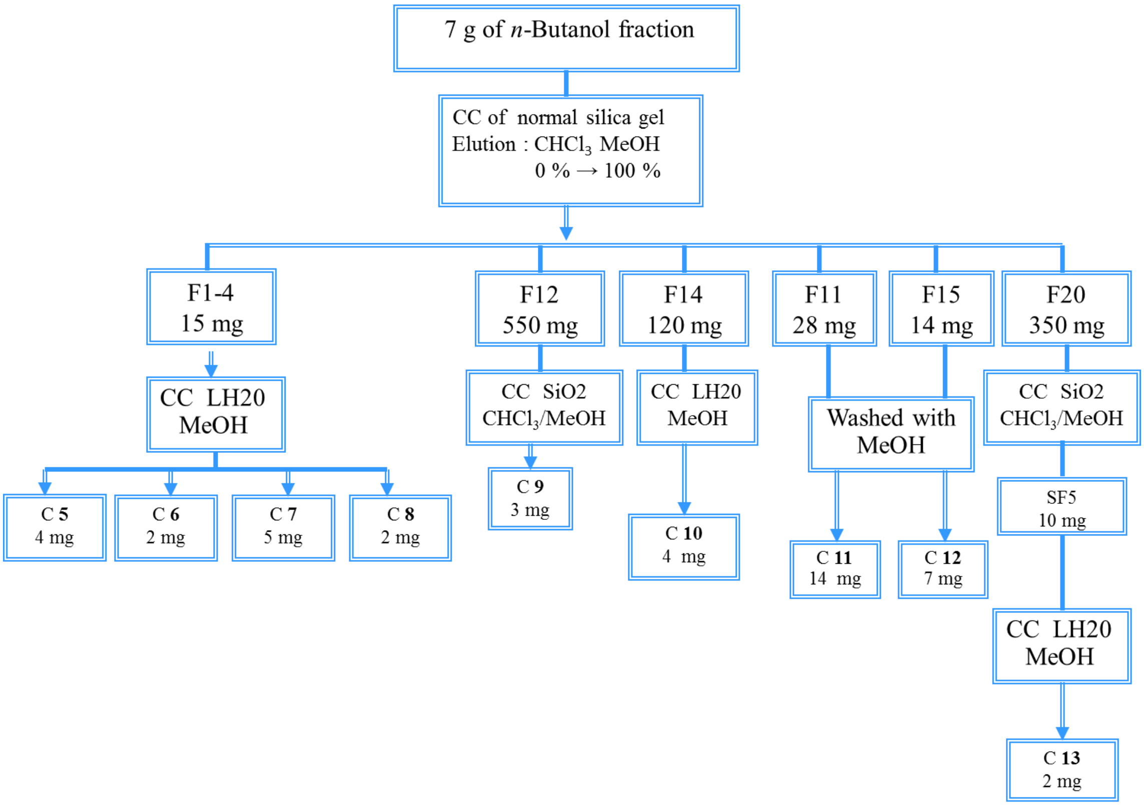

Figure S2. Scheme of the *n*-butanol fraction purification
